# Genome data uncover four synergistic key regulators for extremely small body size in horses

**DOI:** 10.1186/s12864-018-4877-5

**Published:** 2018-06-25

**Authors:** Julia Metzger, Janina Rau, Fanny Naccache, Laura Bas Conn, Gabriella Lindgren, Ottmar Distl

**Affiliations:** 1 Institute for Animal Breeding and Genetics, University of Veterinary Medicine Hannover, 30559 Hannover, Germany; 2 Unit of Reproductive Medicine of the Clinics, University of Veterinary Medicine Hannover, 30559 Hannover, Germany; 3 Department of Animal Breeding and Genetics, Swedish University of Agricultural Sciences, 75007 Uppsala, Sweden

**Keywords:** Body size, Horse, Miniature, ROH, Next generation sequencing, Synergism, Pony

## Abstract

**Background:**

Miniature size in horses represents an extreme reduction of withers height that originated after domestication. In some breeds, it is a highly desired trait representing a breed- or subtype-specific feature. The genomic changes that emerged due to strong-targeted selection towards this distinct type remain unclear.

**Results:**

Comparisons of whole-genome sequencing data from two Miniature Shetland ponies and one standard-sized Shetland pony, performed to elucidate genetic determinants for miniature size, revealed four synergistic variants, limiting withers height to 34.25 in. (87 cm). Runs of homozygosity regions were detected spanning these four variants in both the Miniature Shetland ponies and the standard-sized Shetland pony. They were shown to be characteristic of the Shetland pony breed, resulting in a miniature type under specific genotypic combinations. These four genetic variants explained 72% of the size variation among Shetland ponies and related breeds. The length of the homozygous regions indicate that they arose over 1000 years ago. In addition, a copy number variant was identified in *DIAPH3* harboring a loss exclusively in ponies and donkeys and thus representing a potential height-associated variant.

**Conclusion:**

This study reveals main drivers for miniature size in horses identified in whole genome data and thus provides relevant candidate genes for extremely short stature in mammals.

**Electronic supplementary material:**

The online version of this article (10.1186/s12864-018-4877-5) contains supplementary material, which is available to authorized users.

## Background

In the course of evolution, body size has been proven to be an essential parameter for species survival. Larger individuals do not necessarily exhibit an evolutionary advantage and may instead show shorter long-term survival than extremely small individuals, which are characterized by more rapid development and lower energy and metabolic demands [1–3]. However, domestication alters such natural survival requirements, with the focus instead shifting to desired phenotypic traits [4]. Targeted human selection of horses was directed on performance and conformational qualities, resulting in more specialized breeds, which are often characterized by a defined maximum height at the withers [4–6]. One of the smallest breeds worldwide, the Shetland pony, emphasizes the evolutionary advantage of small body size, surviving as extremely robust individuals on the Scandinavian tundra and later on the Shetland Islands [7]. The versatile application of the Shetland pony as a workhorse in coal mines until the 1990s, as a sports pony and as a companion animal has resulted in the subdivision of these ponies into the miniature type, with a maximum height of 87 cm (34.25 in.) at the withers, and a standard-sized type with a height up to 107 cm (42.13 in.) [7, 8].

Previous investigations of potential size-determining genes in horses, including *ligand dependent nuclear receptor corepressor like (LCORL), zinc finger protein 406 (ZFAT), LIM and SH3 protein 1 (LASP1)* and *ankyrin repeat domain 1 (ANKRD1),* have not explained the differences in withers height between Miniature and standard-sized Shetland ponies [9–11]. Shetland ponies were shown to be fixed for the pony-size-associated genotype of a variant located in a potential transcription factor binding site of *LCORL,* a primary regulator of withers height in horses [9, 12]. *LCORL* was found to be equally high expressed in Shetland ponies of all sizes [12]. Genome-wide association analyses of withers height within a small Shetland pony cohort revealed a significant peak in *high mobility group AT-hook 2 (HMGA2)* region [13]*.* A non-synonymous *HMGA2* variant in a gap in the reference genome EquCab2.0 has been indicated to segregate among Miniature Shetland ponies, standard-sized Shetland ponies and other pony breeds but does not explain the sharp distinction between miniature and standard sizes [13]. Further size-limiting variants in horses have yet to be identified. Thus, we investigated Miniature Shetland ponies compared to standard-sized Shetland ponies for signatures of selection, as well as withers height determining candidate variants, as an excellent model for miniature size development in horses.

## Results

### Whole-genome sequencing data

Mapping of whole-genome sequencing data from two Miniature Shetland ponies and one standard-sized Shetland pony with stringent quality parameters revealed a coverage of 16.71X-25.59X, an error rate of 1.68e-02 to 4.46e-03 and an average quality of 31.6–36.3 (Additional file 1). Variant calling along with further 17 controls of different horse populations, six Przewalski horses, five Scythian stallions from Berel’ and one Donkey resulted in 14,756,113 SNPs and 1,194,044 INDELs.

### Shetland pony specific selection signatures

To identify potential signatures of selection, runs of homozygosity (ROH) and Fst detection was performed for 239,475 filtered confident SNPs in the three Shetland ponies tested. ROH analysis resulted in 460 shared ROH regions harboring 1494 genes, which covered in total 4.355% of the genome (Additional file 2). One shared ROH region was identified spanning *HMGA2*, which was shown to harbor a body size-associated variant (c.83G > A) in a gap of the reference genome EquCab2.0 [13]. A large proportion of the genes detected in shared ROH regions was functionally classified into biological processes affecting cellular (GO:0009987), metabolic (GO:0008152) and developmental (GO:0032502) effects (Table 1).

**Table 1:** Functional annotation of shared ROH regions

PANTHER gene ontology terms	Genes in shared ROH regions in Shetland ponies (%)
cellular component organization or biogenesis (GO:0071840)	4.90
cellular process (GO:0009987)	29.70
localization (GO:0051179)	7.00
reproduction (GO:0000003)	2.10
biological regulation (GO:0065007)	6.40
response to stimulus (GO:0050896)	6.50
developmental process (GO:0032502)	7.50
multicellular organismal process (GO:0032501)	5.40
locomotion (GO:0040011)	0.20
biological adhesion (GO:0022610)	1.70
metabolic process (GO:0008152)	24.90
growth (GO:0040007)	0.10
immune system process (GO:0002376)	3.50

Results from PANTHER gene list analysis for biological processes in human orthologues of genes found in analysis for shared ROH regions in the two Miniature Shetland ponies and on standard sized Shetland pony are shown. The proportion of gene hits against total number of process hits was computed for each gene ontology term

**Fig. 1:**
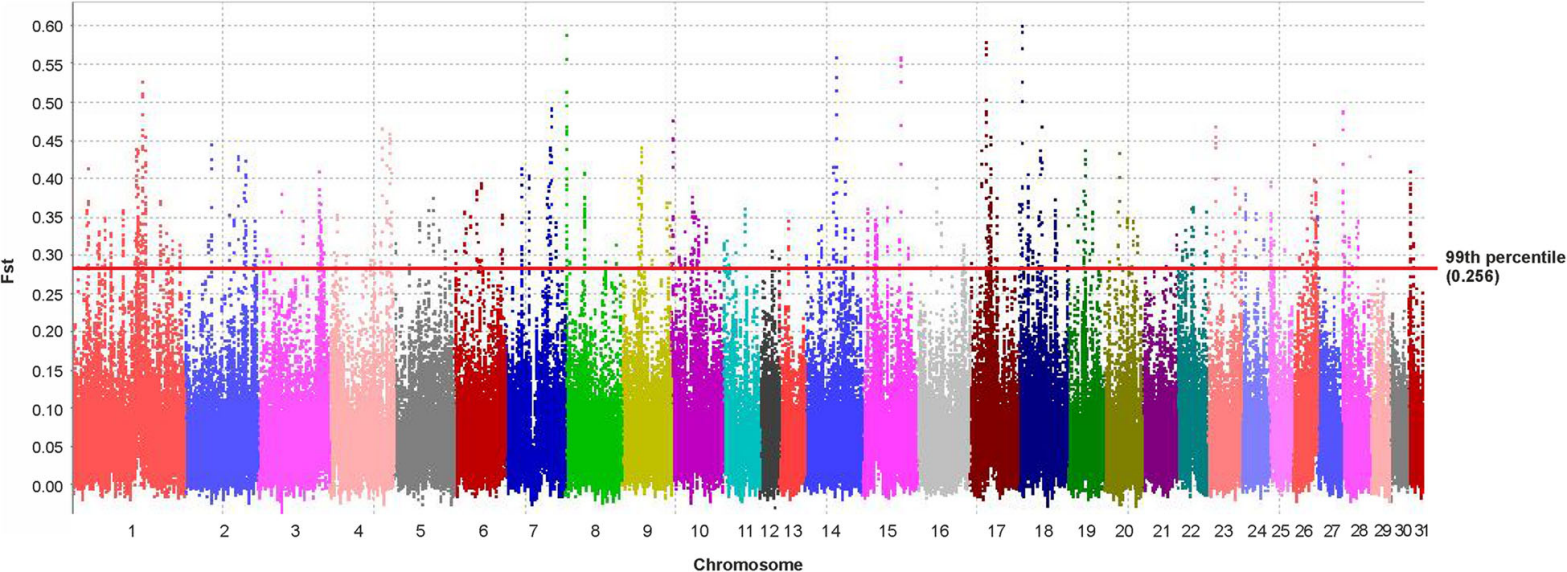
Pairwise Fst between three Shetland ponies and a pool of 24 equids. The individual chromosomes are plotted against Fst detected in windows of 50,000 bp

Further estimation of the population differentiation (Fst) between Shetland ponies and a pool of 24 equids revealed the highest Fst in the 99th percentile (Fst ≥ 0.256) in 2240 regions (Fig. 1). The maximum peaks were observed on ECA17 at 28,480,000-28,530,000 bp (Fst = 0.579), on ECA18 at 7,840,000-7,910,000 bp (Fst = 0.571, 0.600, 0.593) and on ECA8 at 1,620,000-1,670,000 bp (Fst = 0.588).

Overlapping results of shared ROHs and the highest 1% Fst revealed in total 439 regions in the intersection (Additional file 3). High signals with maximum Fst of 0.457 were detected within shared ROHs on ECA1 in the region of 104,360,962-121,600,359 bp, harboring 37 genes including *disintegrin-like and metalloprotease with thrombospondin type 1 motif 17 (ADAMTS17), myotubularin related protein 10 (MTMR10), transient receptor potential cation channel subfamily M member 1 (TRPM1) and myosin IXA (MYO9A)* among others. In addition, an overlap was identified on ECA2 at 88,383,449-88,557,987 bp in the region of *FRAS1 related extracellular matrix 3 (FREM3), SWI/SNF related matrix associated actin dependent regulator of chromatin subfamily a member 5 (SMARCA5)* and *growth factor receptor bound protein 2-associated protein 1 (GAB1),* on ECA9 at 32,602,529-33,046,777 bp in the region of *syntrophin gamma 1 (SNTG1)* and on ECA19 at 28,408,427-28,686,627 bp harboring *osteocrin (OSTN)* and *Urotensin 2B (UTS2B).*

### Identification of genetic variants in ROH regions

In total, 1364 genetic variants with predicted high or moderate effects were identified in shared Shetland pony ROH regions (Additional file 4). SIFT predictions revealed 216 potentially deleterious, 816 potentially tolerated effects and 905 further effects which could not be estimated by SIFT.

Filtering for variants with exclusively homozygous mutant genotypes in the Shetland ponies resulted in only two single nucleotide variants (SNVs), NC_009144.2:g.105258161C > A located in *ADAMTS17* and NC_009162.2:g.28594461G > A located in *OSTN*, which have already been found in high Fst regions overlapping with ROH regions. Effect predictions suggested the missense variant in *ADAMTS17* (NC_009144.2:g.105258161C > A, ss#2137497758) to be deleterious (0.00, SIFT) and probably damaging (1.000, PolyPhen-2) for both transcripts ENSECAT00000000777 and ENSECAT00000000726. The missense and splice region variant NC_009162.2:g.28594461G > A (ss#2137497759) located in *OSTN* was predicted to be tolerated (0.07; SIFT) as well as probably damaging (0.999; PolyPhen-2). Functional classification of *ADAMTS17* and *OSTN* using DAVID annotation tool revealed both genes to be related with bone growth processes (Additional file 5).

**Fig. 2:**
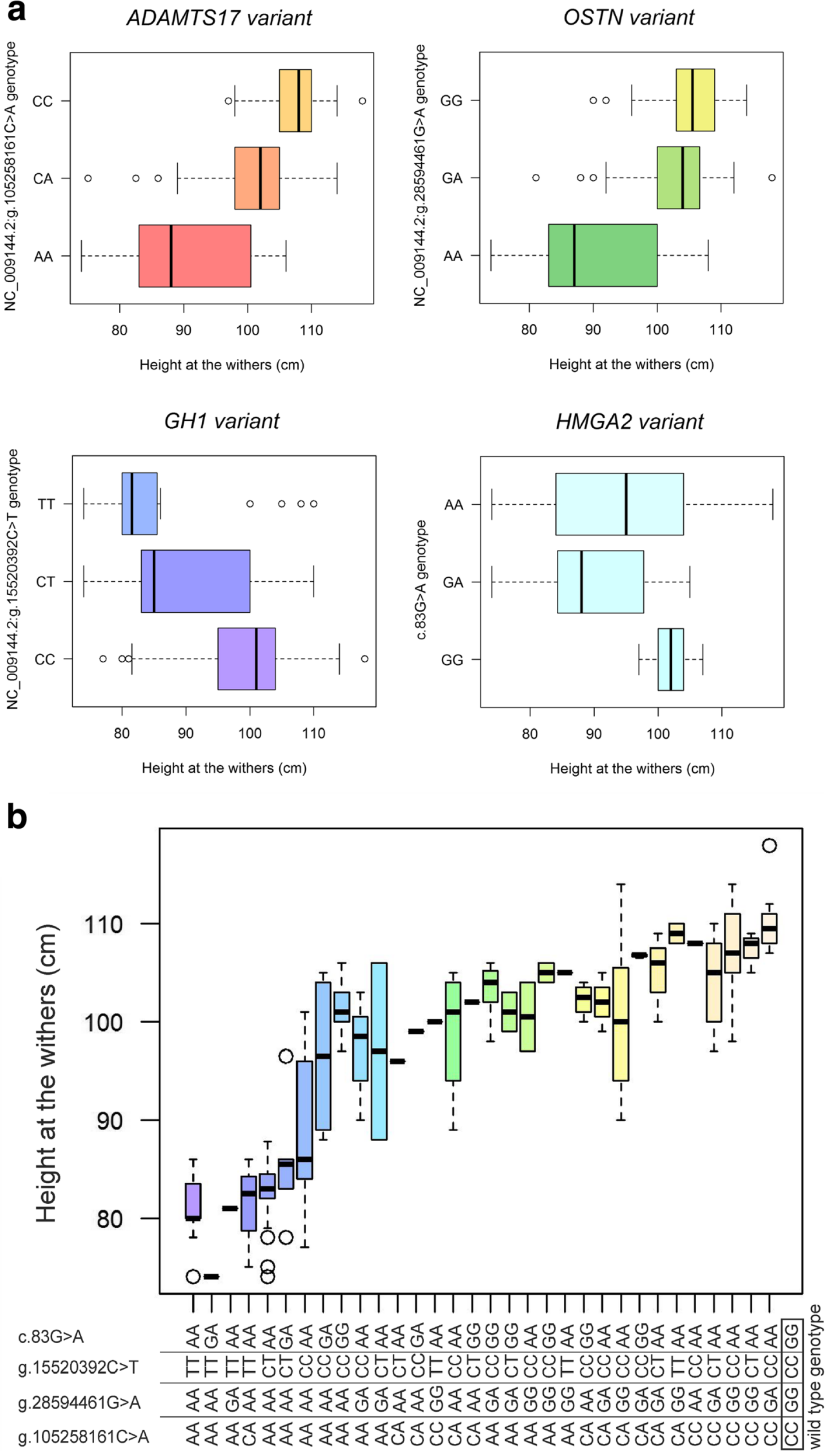
Genotype distribution of height associated variants. The withers height of the investigated Shetland ponies and related breeds is compared with the variant genotypes of *ADAMTS17*, *OSTN*, *GH1* and *HMGA2* (**a**). General linear model analysis confirmed a significant association of variant genotypes with height at the withers. A considerably lower withers height can be seen in ponies with a homozygous mutant genotype for all four variants. Comparative evaluation of all four genotypes shows a powerful synergistic effect (**b**). None of the horses with homozygous mutant genotypes of all variants or homozygous mutant genotypes of three variants plus a heterozygous genotype of the fourth variant shows a withers height higher than 87 cm

In addition, functional analysis of all 216 variants, which were estimated to be deleterious, resulted in nine variants in nine genes (including *ADAMTS17*) involved in bone development, muscle development or growth related phenotypes. One variant on ECA25 at 940,044 bp could not be validated by Sanger sequencing. Further genotyping of the other eight variants in the validation sample-set of 255 equids revealed two variants located in *ADAMTS17* (ECA1 at 105,258,161 bp) and in *growth hormone 1 (GH1;* ECA11 at 15,520,392 bp; PRJEB24630), which could be exclusively found in Shetland and Miniature Shetland ponies as well as German Classic ponies and American Miniature Horses (Table 2). In addition, the *OSTN*-variant could only be found in Shetland ponies, Miniature Shetland ponies, American Miniature Horses and in the Lewitzer pony. Validation of these three variants in the refinement-sample-set of different Shetland and Miniature Shetland ponies confirmed a significant association with height at the withers (Fig. 2a, Table 3). Furthermore, a significant association with height at the withers could also be confirmed for the additionally genotyped and potentially height-associated *HMGA2*-variant c.83G > A found in a ROH region. Consideration of the *ADAMTS17*, *OSTN*, *GH1* and *HMGA2* variant together revealed that none of the ponies that were homozygous for the mutant allele in all four variants or homozygous for the mutant allele in three variants and heterozygous in one variant exhibited a withers height greater than 87 cm (34.24 in.) (Fig. 2b). However, four Miniature Shetland ponies and two American Miniature Horses were also of miniature size with homozygous mutant genotypes in three variants and a homozygous wild type genotype in *GH1*-variant, or with two heterozygous genotypes in *GH1* and *HMGA2*-variant but mutant genotypes in the other two variants.

**Table 2:** Validation of selected variants in ROH regions

	Minor allele frequency								
	n	ECA1 105,258,161	ECA9 36,855,980	ECA11 15,475,283	ECA11 15,520,392	ECA11 15,849,785	ECA19 28,594,461	ECA28 7,301,507	ECA28 7,329,353
Minor allele		A	A	A	T	G	A	T	G
Breed/ population									
American Miniature Horse	5	0.10	0.00	0.00	0.70	0.00	0.40	0.00	0.30
Arabian Thoroughbred	8	0.00	0.00	0.00	0.0	0.00	0.00	0.00	0.00
German Classic Pony	23	0.17	0.04	0.00	0.09	0.00	0.20	0.09	0.13
Connemara pony	4	0.00	0.25	0.00	0.00	0.00	0.00	0.00	0.13
Criollo horse	3	0.00	0.50	0.00	0.00	0.00	0.00	0.17	0.00
Duelmen horse	8	0.00	0.00	0.00	0.00	0.00	0.00	0.00	0.00
Donkey	4	0.00	0.50	0.00	0.00	0.00	0.00	0.00	0.00
Fell pony	1	0.00	0.00	0.00	0.00	0.00	0.00	0.00	0.00
Friesian horse	6	0.00	0.00	0.00	0.00	0.00	0.00	0.00	0.00
German Riding Pony	9	0.00	0.00	0.00	0.00	0.00	0.00	0.00	0.00
Hackney pony	1	0.00	0.00	0.00	0.00	0.00	0.00	0.00	0.00
Haflinger	9	0.00	0.17	0.00	0.00	0.00	0.00	0.00	0.44
Hanoverian	25	0.00	0.00	0.00	0.00	0.00	0.00	0.00	0.06
Holsteiner	1	0.00	0.00	0.00	0.00	0.00	0.00	0.00	0.00
Icelandic horse	9	0.00	0.00	0.00	0.00	0.00	0.00	0.00	0.22
Austrian Coldblood	6	0.00	0.00	0.00	0.00	0.00	0.00	0.00	0.17
Lewitzer pony	7	0.00	0.14	0.00	0.00	0.00	0.14	0.00	0.00
Lusitano	4	0.00	0.00	0.00	0.00	0.00	0.00	0.00	0.00
Miniature Shetland pony	44	0.86	0.23	0.00	0.46	0.00	0.99	0.18	0.34
New Forest pony	1	0.00	0.50	0.00	0.00	0.00	0.00	0.00	0.50
Noriker	1	0.00	0.00	0.00	0.00	0.00	0.00	0.00	0.50
Norwegian Fjord Horse	5	0.00	0.20	0.00	0.00	0.00	0.00	0.00	0.10
Oldenburger	1	0.00	0.00	0.00	0.00	0.00	0.00	0.00	0.00
Peruvian Paso	4	0.00	0.00	0.00	0.00	0.00	0.00	0.00	0.00
Przewalski	2	0.00	0.00	0.00	0.00	0.00	0.00	0.00	0.00
Rhenish German Coldblood	9	0.00	0.33	0.00	0.00	0.00	0.00	0.00	0.06
Schleswig Coldblood	1	0.00	0.00	0.00	0.00	0.00	0.00	0.00	0.00
Black Forest Horse	5	0.00	0.20	0.00	0.00	0.00	0.00	0.00	0.10
Swedish Warmblood	1	0.00	0.00	0.00	0.00	0.00	0.00	0.00	0.00
Shetland pony	15	0.70	0.03	0.00	0.40	0.00	0.50	0.43	0.20
Shetland pony-intermixes	4	0.00	0.00	0.00	0.00	0.00	0.00	0.13	0.00
Shire Horse	1	0.00	0.50	0.00	0.00	0.00	0.00	0.00	0.00
Sorraia	2	0.00	0.00	0.00	0.00	0.00	0.00	0.00	0.00
Standardbred	8	0.00	0.13	0.00	0.00	0.00	0.00	0.00	0.00
Trakehner	1	0.00	0.00	0.00	0.00	0.00	0.00	0.00	0.00
Welsh Cob (Section D)	12	0.00	0.04	0.00	0.00	0.00	0.00	0.00	0.04
Welsh Mountain Pony (Section A)	5	0.00	0.10	0.00	0.00	0.00	0.00	0.20	0.00

In total, seven variants derived from filtering for variants with deleterious effects and one variant with an exclusively homozygous mutant genotype in all three Shetland ponies were validated in 255 equids of 36 populations. The number of genotyped individuals and minor allele frequency for each population is shown

**Table 3:** General linear model analysis for body size

ECA	Gene	Polymorphism	R-Square	F-value	P-value	Genotype	LSMean (height, cm)	Standard error
1	*ADAMTS17*	NC_009144.2:g.105258161C > A	0.34	61.51	2.7089E-22	A/A	91.27	0.68
						C/A	100.39	1.26
						C/C	107.14	1.41
6	*HMGA2*	c.83G > A	0.12	18.81	1.47E-7	G/G	102.33	1.38
						G/A	89.82	2.97
						A/A	93.80	0.73
19	*OSTN*	NC_009162.2:g.28594461G > A	0.40	78.57	5.646E-27	A/A	90.36	0.66
						G/A	102.99	1.11
						G/G	105.17	1.36
11	*GH1*	NC_009154.2:g.15520392C > T	0.22	33.91	1.0714E-13	C/C	98.64	0.72
						C/T	90.22	1.26
						T/T	84.90	1.89

The genotype effects on height at the withers are shown for all four SNV/SNPs validated in 243 Shetland ponies and related breeds whose height at the withers was measured

The size of ROH regions harboring the four height-associated variants in each Shetland pony (0.28–3.34 Mb) led to the estimation that miniature size emerged up to 120–180 generations and thus 1200–1800 years ago (Table 4).

**Table 4:** Tracing back regions under selection

	Shetland pony 1 (SRX1976860)			Shetland pony 2 (ERX947604)			Shetland pony 3 (ERX947605)		
Gene in ROH region	SIZE of ROH region (Mb)	Number of generations ago	Number of years ago	SIZE of ROH region (Mb)	Number of generations ago	Number of years ago	SIZE of ROH region (Mb)	Number of generations ago	Number of years ago
*ADAMTS17*	2.522687	19.82	198.20	1.062162	47.07	470.74	3.342086	14.96	149.61
*GH1*	0.635954	78.62	786.22	2.837501	17.62	176.21	0.492062	101.61	1016.13
*OSTN*	0.278201	179.73	1797.26	0.406642	122.96	1229.58	0.413306	120.98	1209.76
*HMGA2*	1.498057	33.38	333.77	1.945192	25.70	257.04	1.118224	44.71	447.14

The size of ROH regions in the four height-associated variants detected in whole-genome sequencing data for each Shetland pony is shown. The number of generations ago (1/2c) and the number of years were estimated for a generation interval of 10 years

### Copy number detection

Relative copy number variant (CNV) detection in paired case-control analyses resulted in 390 CNVs (Table 5) compared to the Hanoverian group, 2398 CNVs in comparison with the Thoroughbred horses, 981 CNVs compared to non-breeds and 593 CNVs compared to other breeds (Additional file 6). The intersection of all these detection results revealed in total 97 potential Shetland pony-specific CNVRs composed of 91 losses and 6 gains. The size of these CNVRs ranged from 121 bp to 9427 bp with an average size of 2689 bp (Additional file 7). In total, 14 genes were identified in 18 CNVRs for which 13 human orthologue genes were found (Additional file 8). Functional analysis for the involvement of these genes in biological processes revealed a high enrichment in cellular processes (31.3%, GO:0009987), response to stimulus (18.8%, GO:0050896), immune system processes (18.8%, GO:0002376), biological adhesion (18.8%, GO:0022610) and also in biological regulation (6.3%, GO:0065007) and localization (6.3%, GO:0051179).

**Table 5:** CNV detection results

Cases	Cases: Sequence Read Archive ID	Controls	Controls: Sequence Read Archive ID	Number of CNVs (loss)	Number of CNVs (gain)
Shetland ponys	SRX1976860, ERX947604, ERX947605	Control group 1 (three Hanoverian)	SRX389480, SRX389477, SRX1131705	253	137
Shetland ponys	SRX1976860, ERX947604, ERX947605	Control group 2 (two Arabian, one Thoroughbred)	SRX389472, SRS431663, SRX396629	860	1538
Shetland ponys	SRX1976860, ERX947604, ERX947605	Control group 3 (Duelmen horse, Sorraia, Przewalski)	SRX384479, SRX389475, SRS441443	329	652
Shetland ponys	SRX1976860, ERX947604, ERX947605	Control group 4 (Saxon-Thuringian Heavy Warmblood, Marwari, Standardbred)	SRX1131818, SRX535352, SRS438330	284	309
Intersection of all groups				91	6

Number of CNVs identified in four paired case-control analyses are shown. The intersection of these detection results revealed 97 potential Shetland pony-specific CNV regions

**Fig. 3:**
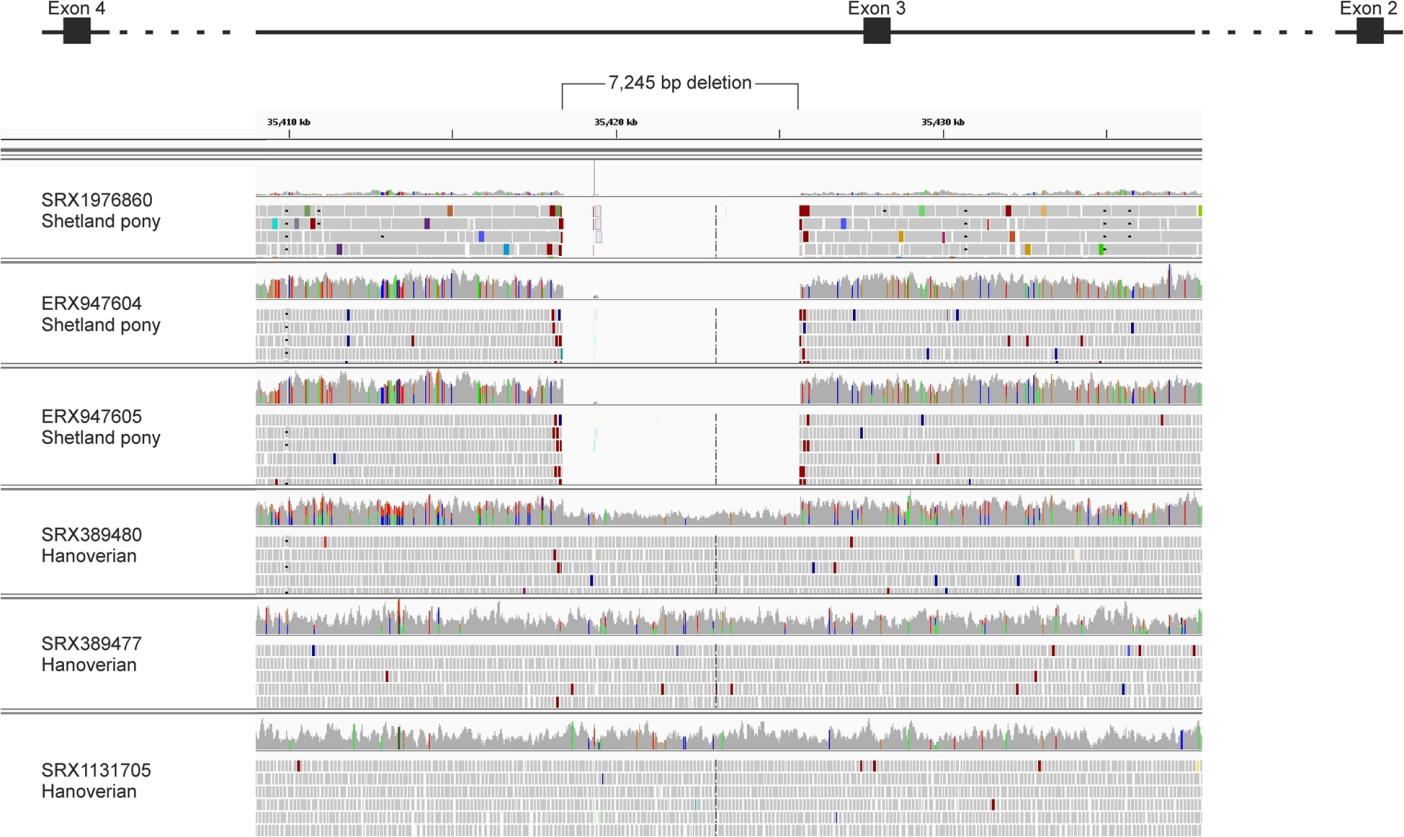
Shetland pony specific deletion located in *DIAPH3*. Integrated Genomics Viewer (IGV) shows bam-files of all three Shetland ponies derived from whole-genome sequencing analysis harbouring a deletion of an estimated size of 7245 bp. In contrast, all control horses reveal an insertion in this region (exemplary display of three Hanoverian horses)

**Fig. 4:**
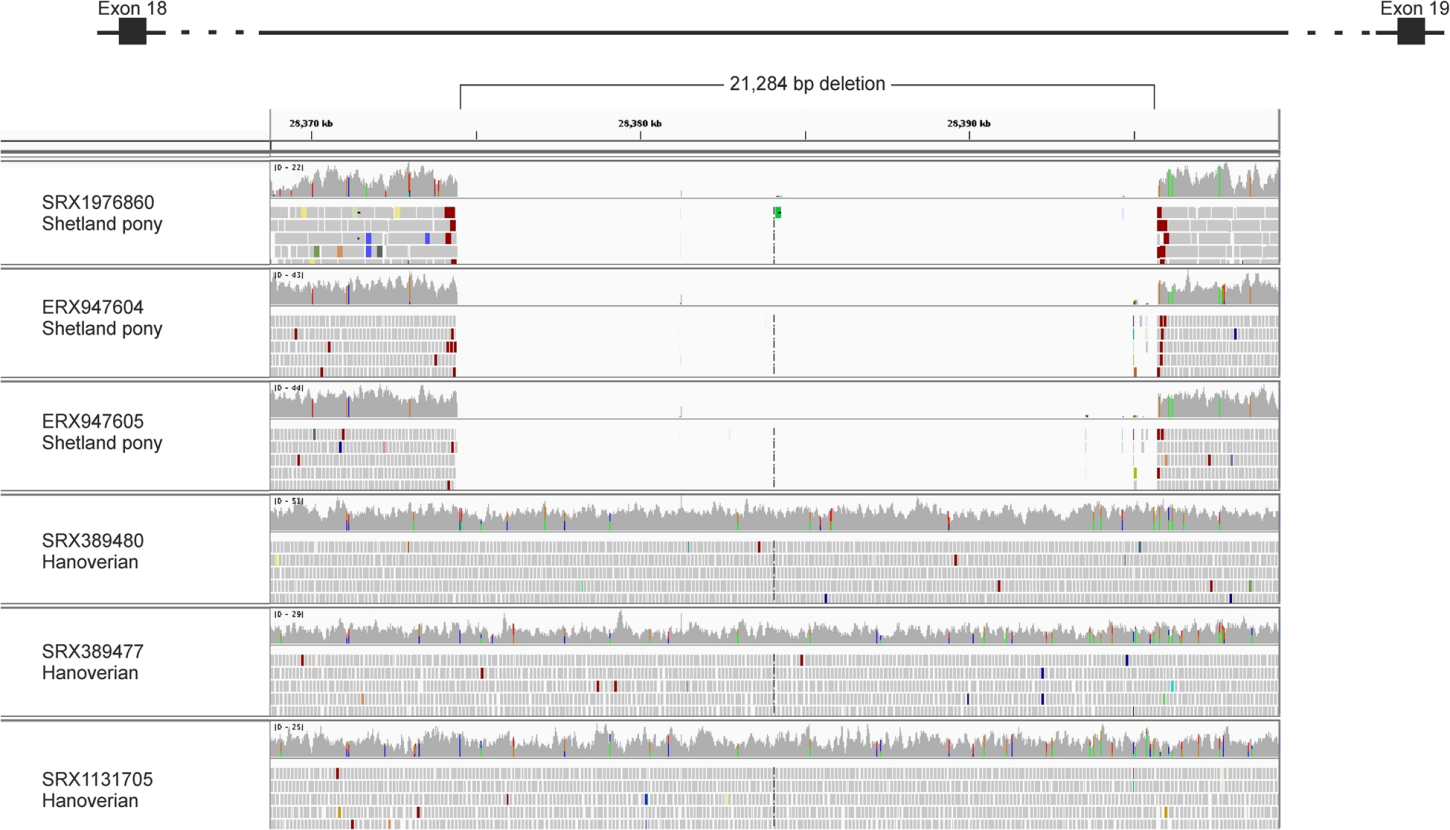
Shetland pony specific deletion located in *VWA8*. Integrated Genomics Viewer (IGV) shows bam-files of all three Shetland ponies derived from whole-genome sequencing analysis harbouring a deletion of an estimated size of 21,284 bp. In contrast, all control horses reveal an insertion in this region (exemplary display of three Hanoverian horses)

Ten CNVRs were located in exonic regions, whereas eight could be exclusively found in intronic regions. Evaluation of all CNVRs located in genes using the integrative genomics viewer (IGV) revealed two homozygous deletions with definable breakpoints in all three Shetland ponies, which could not be identified in any other investigated bam-file. One of these deletions was located on ECA17 at 35,418,404-35,425,648 bps in the region of *diaphanous related formin 3 (DIAPH3)* (Fig. 3). The other deletion spanned three detected CNVRs on ECA17 from 28,374,441 to 28,395,725 bps in the region of *von willebrand factor A domain containing 8 (VWA8;* Fig. 4*).* In addition, further ten CNVRs suggested a heterozygous loss or gain in the Shetland ponies whereas the remaining four CNVRs could be found to be homozygous deletions only in two Shetland ponies.

**Fig. 5:**
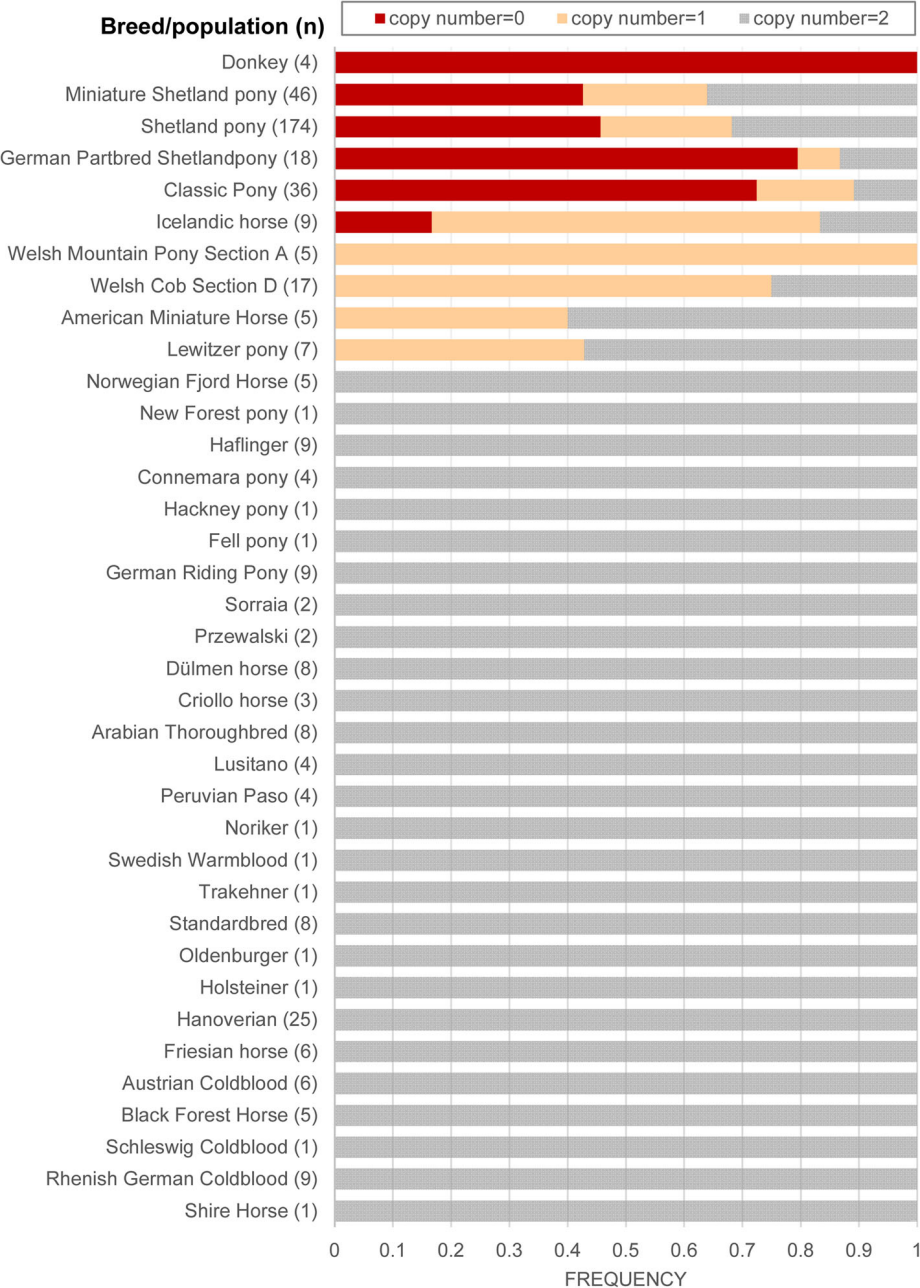
Frequency of *DIAPH3* deletion in horse breeds/ populations. CNV detection in the whole validation sample set reveals copy numbers of 1 or 0 exclusively in the Shetland ponies and related breeds as well as in the Icelandic horse, Lewitzer, Welsh and Donkey

Both homozygous deletions could be validated by multiplex PCR. The deletion located in *DIAPH3* was only found in two of the investigated Shetland ponies and in all three Miniature Shetland ponies but not in any other investigated horse sample. In contrast, the deletion in *VWA8* could be detected in two Shetland ponies, all three Miniature Shetland ponies and all investigated Rhenish German Coldblood horses as well. Due to its exclusive occurrence in the genotyped Shetland ponies, the deletion located in *DIAPH3* was further validated by relative real-time PCR in the validation sample-set and the refinement-sample-set of 447 horses and ponies. The 7245-bp deletion located in intron 3 of *DIAPH3* could be shown to harbor a loss exclusively detected in Shetland ponies and related breeds as well as in the Icelandic horse, Lewitzer, Welsh and Donkey but not in any other tested horse breed (Fig. 5).

**Fig. 6:**
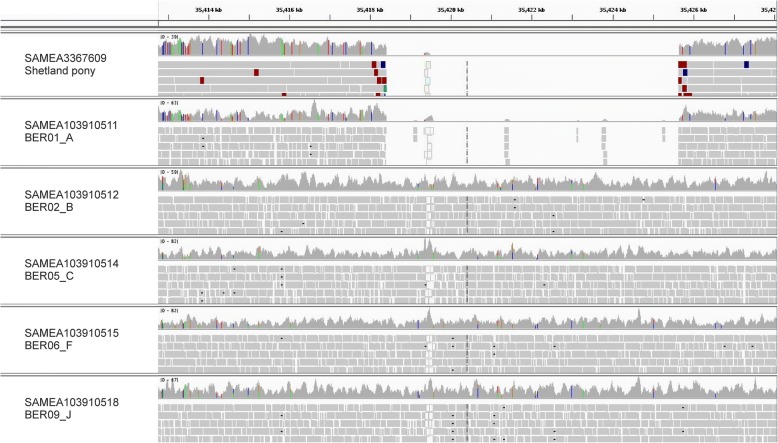
Pony-specific deletion found in *DIAPH3*. Graphical representation of a region of *DIAPH3* revealing a 7245 bp loss in intron 3, which can be observed in the Shetland pony and in one ancient Berel’ horse

It was also detected to harbor a loss in an ancient sample of a Scythian stallion from Berel' (Fig. 6). The distribution of losses and gains revealed no significant association with height at the withers or cannon bone circumference (*P* = 0.19373; *P* = 0.78549).

### Correlation in-between candidate variants for body size

Investigations of potential additive effects of the four detected missense variants on the development of body size suggested the three novel SNVs located in *ADAMTS17*, *OSTN* and *GH1* to explain 61% of the variance. An even higher R-square could be ascertained for all three SNVs and the SNP located in *HMGA2* explaining 72% of the variance in withers height among 243 investigated Swedish and German Shetland ponies and individuals of related breeds, including German Classic ponies, German Partbred Shetland ponies and American Miniature Horses. In direct investigation of all four SNV/SNP genotypes as one complex genotype, the R-square was even higher (0.80). Joint GLM analysis of all four variants revealed a significant effect for each variant contributing to the total synergistic additive effect on withers height (Additional file 9). The differences in-between the genotypes remained at the same levels. The sum of the additive effects resulted in an estimation of a size reduction of 18.48 cm in ponies with the homozygous mutant genotype in comparison to ponies with homozygous wild type genotype. In addition, the *OSTN* variant showed a low dominance effect. In contrast, the CNV harboring *DIAPH3* did not contribute to the additive effect. The inter-variant allele count correlations (r^2^) for all four SNVs/SNPs genotyped in Shetland ponies and Shetland pony related breeds revealed little linkage in-between the individual variant loci (Table 6). As already seen in general mixed model analysis for the individual variants, the variants located in *OSTN* and *ADAMTS17* possessed the highest effects on height at the withers (40 and 34%). Network analysis predicted both *ADAMTS17* and *HMGA2* to be co-expressed with *growth hormone receptor (GHR),* physically interacting with *GH1*. Furthermore, a direct co-expression of *ADAMTS17* with *GH1* was also predicted (Additional file 10). In addition, *HMGA2* was found to physically interact with *SMAD9*, which was predicted to interact with *DIAPH3*. Furthermore, a similar additive effect of the four variants was assessed for cannon bone circumference in 52 Shetland ponies and Shetland pony related breeds whose cannon bone measures were available. This finding was supported by the identification of a high correlation in-between height at the withers and cannon bone circumference (0.74). Three variants in *HMGA2*, *OSTN* and *GH1* were significantly associated with cannon bone circumference (Additional file 11). *ADAMTS17* did not reach the level of significance but was estimated to explain in total 60% of the variance together with the other three variants. The influence of *HMGA2* variant on cannon bone circumference was suggested to be quite high (52%).

**Table 6:** Linkage disequilibrium for height-associated variants

	*ADAMTS17* (ECA1) g.105258161C > A	*OSTN* (ECA19) g.28594461G > A	*GH1* (ECA11) g.15520392C > T	*DIAPH3* (ECA17) g.35425648_35418404del	*HMGA2* (ECA6) *HMGA2*:c.83G > A
g.105258161C > A		0.0711316	0.00250835	0.0495211	0.394906
g.28594461G > A	0.0711316		0.0665219	0.0384475	0.0104857
g.15520392C > T	0.00250835	0.0665219		0.00746066	0.0127573
g.35425648_35418404del	0.0495211	0.0384475	0.00746066		0.0213281
*HMGA2*:c.83G > A	0.394906	0.0104857	0.0127573	0.0213281	

Inter-variant allele count correlations (r^2^) are shown for all five variants genotyped in Shetland ponies and Shetland pony related breeds

## Discussion

Three novel missense variants located in *ADAMTS17, GH1* and *OSTN* were found to be associated with withers height and were validated as being exclusive to Shetland ponies and Shetland pony-related breeds. In addition, a further ROH spanning *HMGA2*, confirmed the body size-associated c.83G > A variant in Shetland ponies [13]. Thus, these variants are suggested to be derived from the Shetland pony breed. We assume that all four size-associated variants were located in ROH regions in the two Miniature Shetland ponies as well as the standard-sized Shetland pony due to the strong-targeted selection in both directions for miniature as well as standard sizes [13]. The identification of the variants in *ADAMTS17* and *OSTN* in regions with maximum Fst peaks overlapping with ROH regions confirmed the previous detection of a potential selection signature in the *ADAMTS17* region in Shetland ponies [14].

We propose that the identified four variants characterizing the Shetland pony breed, result in the development of a miniature type under specific genotype combinations and therefore originated in the Shetland pony itself. The most likely explanation is a synergistic action of these four variants as negative key regulators for growth. In humans, both *ADAMTS17* and *HMGA2* are predicted to be co-expressed with the *GHR*, which physically interacts with *GH1* [15, 16]. These findings are consistent with the reported high impacts of *GH1, OSTN* and *HMGA2* on chondrocyte proliferation and osteoblast secretion [17–20]. Mutant *aggrecanase* gene, *ADAMTS17,* has been shown to affect human height, causing an abnormally short stature and shorted extremities in affected patients [21, 22]. In addition, *GH1* was considered to harbor a height-associated variant in African populations [23]. The roles of these genes in bone morphology and composition indicate that the additive effect of the four potentially deleterious variants could severely inhibit bone formation, resulting in the desired miniature type. Thus, we suggest that these genes should be considered jointly as candidates for main drivers of miniature size in horses.

Our investigations of Shetland ponies and related breeds showed that none of the ponies with homozygous mutant genotypes in all four variants or only one heterozygous genotype exceeded a height at the withers of 87 cm (34.25 in.) and thus confirmed the size-limiting effect of these variants. However, the detection of few cases of miniature-sized Shetland ponies and American Miniature Horses with a homozygous wild type genotype in one variant or two heterozygous genotypes in two variants, suggests that potential further gene effects promoting endochondral ossification were ineffective in these horses. As our study was mainly based on European Shetland ponies, it is unclear, if additional size-limiting variants might have emerged from other non-European miniature breeds. Strong targeted selection of characteristic phenotypes, particularly observed in American Miniature Horses [24], might have promoted further rare variants affecting bone growth.

The origin of selective pressures for miniature size in Shetland ponies presumably dates back 120–180 generations, to approximately 1200–1800 years ago. None of the miniature-size related variants were identified in the ancient genomes of five Scythian stallions from Berel’, which represent early domestic horses from the Iron Age, ~ 2.3 thousand years ago [25]. Thus, as all investigated Scythian horses were observed to harbor the pony-associated *LCORL* genotype [25], we assume that Scythian horses might have been ponies with a withers height likely ranging from > 87 cm to ≤148 cm (> 34.25 to ≤58.27 in.). It confirms our assumption that miniature size occurred after domestication in the course of further development of specific breed types.

Furthermore, this conclusion was supported by the detection of a CNV located in *DIAPH3* harboring a loss exclusively in ponies, donkeys as well as a Scythian horse, indicating that this variant provides early evidence of pony or small-equine-specific features. Although the CNV was not found to contribute to the additive effect of the identified SNPs on withers height in Shetland ponies, its occurrence in small equines suggests a role of this variant in growth related characteristics like skull and body length or shape in which particularly ponies distinguish themselves from larger horse breeds. *DIAPH3* was predicted to physically interact with *SMAD9*, a transcriptional regulator in bone, which also interacts with *HMGA2* in humans [26, 27]. Thus, a loss in *DIAPH3* intron might affect its interplay in this growth cascade. Furthermore, previous investigations on CNVs in horses supported the suggestion that structural variants can have an essential influence on skeletal development [28]. Either losses or gains were identified in growth regulatory genes. In general, the identification of duplications was reported to be more challenging than the identification of deletions [29], as we could observe in our study. Therefore, we cannot exclude the involvement of further structural variation, in particular gains, that might play a role in growth regulation in Shetland ponies.

## Conclusions

Hence, our findings provide four synergistic size-inhibiting variants that limit the withers height of ponies to a miniature size, allowing insight into early horse development and the impact of domestication. In addition, our investigations emphasize the horse to be a perfect model for the investigation of size. The identified candidate genes should be considered jointly as candidates for main drivers of miniature size in mammals.

Knowledge of these variants will allow breeders to improve mating strategies and perform targeted selection for miniature size in horses.

## Methods

### Animals

In total three Shetland ponies were used for whole-genome sequencing data analysis. Data files were derived from previous analysis of one Miniature Shetland pony (SRX1976860 [30]) as well as from a further study of one Miniature Shetland pony and one standard-sized Shetland pony (ERX947604/ ERX947605) [13]. Furthermore, whole-genome sequencing data from 29 equine controls derived from our horse population studies [5, 31] and previous studies on ancient genomes [25, 32] including Scythian stallions from Berel’ were downloaded from Sequence Read Archive (NCBI).

For validation of identified variants in this study, 447 DNA-samples from different equids were available. A subset from these samples of 255 equids was used for validation of all identified height-associated or Shetland pony-specific variants in a first step. This sample set included 44 Miniature Shetland ponies, 15 standard sized Shetland ponies, four Shetland pony intermixes, 23 German Classic ponies, five American Miniature Horses and further 160 controls of 31 different horse populations and four donkeys. Furthermore, those variants that needed further refinement were additionally genotyped in the refinement-sample-set of 192 different Shetland ponies, Miniature Shetland ponies and German Partbred Shetland ponies from Germany and Sweden. Altogether, the height at the withers was measured and documented in 243 adult ponies. In addition, in 52 horses the cannon bone circumference was recorded.

### Whole-genome sequence analysis

Fastq-files derived from whole-genome sequencing of two Miniature Shetland ponies, one Shetland pony and 29 controls were downloaded and trimmed using PRINSEQ v 0.20.4 [33] (dust method with maximum allowed score of 90 and trimming from 5′-end and 3′-end with threshold quality score of 20). Mapping was performed for the individual files to the reference genome EquCab 2.0 (ftp://ftp.ensembl.org) including unknown contigs (ChrUn) using BWA v 0.7.13 [34]. Bam-files were further processed using SAMtools v 1.3.1 [35] for sorting and indexing as well as Picard tools (http://broadinstitute.github.io/picard, v 2.3.0) to mark duplicates. Finally, tools from GATK 3.5 [36] including Realigner Target Creator, Indel Realigner, Base Recalibrator, Base Quality Score Recalibration (BQSR), Unified Genotyper and Variant Annotator were used for variant calling. Variant effects were estimated on basis of SNPEff v 4.1 [37] (EquCab 2.86 database) predictions. Chromosome X and ChrUn were omitted from analysis.

### ROH detection

Filtering was done for confident SNPs in all 32 equids applying a read depth of 3–60 and quality values > 20. ROHs with at least 50 SNPs, a total length greater than or equal to 150 kb and with at least one SNP per 3 kb were identified using PLINK, version 1.07 (http://pngu.mgh.harvard.edu/purcell/plink/). A maximum of ten SNPs with missing genotypes and three heterozygous SNPs were admitted in each window. Consecutive SNPs with a distance of more than 100 kb apart were not allowed in the same ROH. Further identification of ROH regions shared by all three Shetland ponies was done using SAS, version 9.4 (Statistical Analysis System, Cary, NC). These regions were investigated for equine genes and human orthologues performed by Galaxy intersection tool (assembly Sept. 2007/ EquCab 2.0) [38, 39], g:Profiler [40] and PANTHER v 11.1 gene list analysis for biological processes [41].

### Variants in ROH regions

Shared ROH regions in all three Shetland ponies were investigated for variants with high or moderate effects according to SNPEff predictions. Those variants, which have not already been documented in dbSNP [42] database (release 87) and were predicted to be deleterious (SIFT [43]), were chosen for further analysis. In addition, we also investigated SNVs with high or moderate effects according to SNPEff predictions, which could be exclusively found homozygous mutant in the investigated Shetland ponies but homozygous wild type in all modern horse populations, Przewalski horses and the donkey. The five Scythian horses were not included in these filtering criteria due to their unclear phenotypes.

Human orthologue genes of all these filtered variants were functionally classified using DAVID Functional Annotation Tool [44, 45]. All variants located in genes that were predicted to be involved in the development of growth related phenotypes, muscle development or bone development were further selected for validation. To confirm the detected variants, Sanger sequencing was performed in one Shetland pony and one Hanoverian from whole-genome sequencing analysis. Genotyping of all validated variants was performed in 255 equids from the validation-sample-set using Kompetitive Allele Specific PCR (KASP, [46]) assays (LGC Genomics, Middlesex, UK) run on an ABI7300 real-time system (Applied Biosystems, Foster City, CA) (Additional file 12). In addition, potential candidate variants and the *HMGA2* variant c.83G > A were further genotyped in the refinement-sample-set including 192 Shetland ponies. For identifying c.83G > A (*HMGA2*) genotypes, primers (F-CTTCTCTCCTCCTCCTCCTC; R-CGCGTACTGACTTGCTGCTG) were designed for the gap region of the equine reference genome where the SNP was detected [13]. Amplification was performed using Q-solution (Qiagen, Hilden, Germany), buffer, dNTPs and Taq polymerase (MP Biomedicals, Santa Ana, CA, USA) for PCR-master mix and run at an annealing temperature of 61.4 °C for 43 cycles. Final digestion was done at 37 °C for 2 h using HgaI (New England Biolabs, Ipswich, Massachusetts, USA).

General mixed model (GLM) analysis was performed for testing the effects of genotypes and alleles on withers height and also on cannon bone circumference using SAS, version 9.4. In addition, the size of ROH regions spanning candidate variants was documented (hom-files) and used for the computation of the number of generations (1/2c). The number of years was estimated for a generation interval of 10 according to previous suggestions in different horse populations [47–49].

### Fst detection and comparison with ROH regions

Fst between the three Shetland ponies and a pool of control samples of 24 equids (Scythian horses not included) were called in sliding windows of 50,000 bp in 10,000 bp steps using the Fst procedure of the analysis of next generation sequencing data tool ANGSD [50]. Results from Fst analysis were plotted against chromosomes and chromosomal positions. Windows with Fst in the 99th percentile of detection results were investigated for an overlap with shared Shetland pony ROH regions using SAS (version 9.4). Intersections were examined for annotated genes in these regions using Galaxy intersection tool [38, 39].

### Copy number detection

CNVs were detected for whole-genome sequencing data in a read-depth based approach using CNV-seq [51]. The two Miniature Shetland ponies and one standard sized Shetland pony were assigned as cases and further 12 horses of different types (Hanoverian, Thoroughbred, non-breed, others) whose coverage reached 10X were used as controls. Fastq files were mapped to the reference genome EquCab 2.0 masked for repetitive sequences (NCBI).

After mapping, the coverage was computed for the resulting bam files using SAMtools. In total four runs with bam files as input were performed for the generation of best-hit files to reach a balanced distribution of reads in cases and controls. In each run, the three Shetland ponies were assigned as cases compared to three individuals each in control group 1 (three Hanoverian), control group 2 (two Arabian, one Thoroughbred), control group 3 (Duelmen horse, Sorraia, Przewalski) and control group 4 (Saxon-Thuringian Heavy Warmblood, Marwari, Standardbred). Each group was composed of three files which showed a total coverage (sum of three coverages) approximating the total control group coverage (58X). The sliding window size and log2 ratios were calculated using the perl script cnv-seq.pl. We applied a log2-threshold of 0.7, default *P*-value = 0.001 and minimum-windows-required = 10 for annotation of CNVs. CNV detection results were investigated for overlapping CNV regions (CNVR) in all four runs representing CNVR exclusively found in Shetland ponies. A minimum overlap of 50 bp was defined as a CNVR intersection. Further functional analysis for the involvement of genes in overlapping CNVR in biological processes was performed using PANTHER v 11.1 [41]. Visualization of CNVR located in genes found in the intersection results was done applying the integrative genomics viewer (IGV) [52]. All whole-genome sequences, which were not run by CNV-seq were visually investigated for all height-associated CNVR.

For confirmation of CNV detection results two CNVR that showed a homozygous loss in all three Shetland ponies, a multiplex PCR was performed using a forward primer in the proximal region of the deletion and two reverse primers. One of the primers was located in the deletion and the other proximally (Additional file 13).

In total, three Shetland ponies, three Miniature Shetland ponies, three Hanoverian and three Rhenish German Coldblood horses were used for validation. A master mix with both primers was prepared following a standard protocol [12] and run for 95 °C for 5 min, 94 °C for 30 s and an annealing temperature of 58.5 °C for 30 s for 41 cycles.

Furthermore, one of the detected homozygous deletions located in *DIAPH3* was investigated by real-time quantitative PCR in six horse samples derived from whole-genome sequencing analysis and in further 255 equids from the validation sample-set. TaqMan Copy Number assays with primers and probes (Thermo Fisher, Applied Biosystems, Waltham, MA USA) were designed for the CNVR on ECA17 at 35,418,241-35,422,357 bp using the GeneAssis Copy Number Assay design tool (Thermo Fisher).

A VIC labeled assay for the CNVR in *DIAPH3* (VIC-CTCGTAGGCAATGGATTCTATC, 35,422,831-35,422,900 bp) with forward (F-CATCCTTCCACCTTGCCTGAA) an reverse primers (R-CACCTCACCACAACGTCCTT) was applied, whereas primers (F-CGATGCTGGTGCTGAATATGTT, R-GGTCAACTCCCCTCATCTTTAGC) and probe (FAM-TCTTCACTACCTTGGAGAAG, 34,076,957-34,077,058) located in *Glyceraldehyde-3-phosphate dehydrogenase (GAPDH)* on ECA6 labeled with FAM dye served as reference assay. Reactions were run in duplex mode on an ABI7300 real-time system (Applied Biosystems) using Maxima Probe qPCR Master Mix (2X) with ROX Solution (Thermo Scientific) according to standard protocols [28]. Samples from whole-genome sequencing analysis served as controls for the relative quantification method based on the 2-ΔΔCt method [53].

### Correlation in-between candidate variants for body size

Correlations in-between the investigated candidate SNVs and CNVs were calculated using GLM analysis testing the effects of genotypes and alleles using SAS, version 9.4. In addition, predictions of biological networks were detected using GeneMANIA [54].

## Additional files

Additional file 1 Mapping statistics of whole-genome sequencing data. In total 32 samples of equids of different populations were analyzed in this study. Sequence read achieve ID, sequencing parameters and mean coverage are shown. (DOCX 15 kb)

Additional file 2 ROH detection results. This table displays all detected ROH regions shared in whole-genome sequencing data of the two Miniature Shetland ponies and one standard sized Shetland pony. The chromosomal position in EquCab 2.0, the number of SNPs in ROH regions, the size of ROH regions and human orthologue genes are shown. (XLSX 73 kb)

Additional file 3 Comparison of ROH regions with Fst. The highest 1% Fst investigated for ROHs in the same region found in three Shetland ponies. In total 439 overlapping regions were identified. (XLSX 20 kb)

Additional file 4 Results from variant effect prediction analysis. All genetic variants with predicted high or moderate effects in shared Shetland pony ROH regions were evaluated for their effects, potential amino acid change and SIFT predictions. (XLSX 111 kb)

Additional file 5 Functional classification of genes. Variants identified in ROH regions in the two Miniature Shetland ponies and one standard sized Shetland pony, which were estimated to be deleterious (SIFT prediction) were investigated for their genomic positions in genes. In addition, variants, which showed an exclusively homozygous mutant genotype in all three Shetland ponies, were added to the analysis. Human orthologue genes were functionally classified using DAVID Functional Annotation Tool. (XLSX 23 kb)

Additional file 6 Graphical representation of CNV detection results. Plots of log2 ratios of comparative analysis of three Shetland ponies with warmblood control group (a), Thoroughbred control group (b), non-breed control group (c) or “other breeds” control group (d) are displayed. (TIFF 3829 kb)

Additional file 7 Characterization of potential Shetland pony-specific CNV regions. The size of the 97 CNV regions identified in the intersection of all four CNV detection runs for Shetland pony-specific CNVs is shown in base pairs (bp) per chromosome. The number and size of all CNV regions varies among the chromosomes. On eleven chromosomes, no CNV region could be found in the overlapping results. (TIFF 16611 kb)

Additional file 8 Intersection of potential Shetland pony-specific CNV regions (CNVRs). The overlap of four CNV detection results for Shetland pony-specific CNVRs shows 97 CNVR harboring 14 different genes. (DOCX 18 kb)

Additional file 9 Genetic effects for height at the withers. The individual additive effects of all four candidate SNPs and the CNV located in DIAPH3 as well as simultaneous testing results for additive and dominant effects are shown. (DOCX 13 kb)

Additional file 10 Functional interaction network of candidate genes. Both, *ADAMTS17* and *HMGA2* show a co-expression (purple lines) with *GHR*, which is physically interacting (red lines) with *GH1*. Furthermore, *ADAMTS17* is directly co-expressed with *GH1*. All three investigated genes *GH1*, *HMGA2* and *DIAPH3* show an interaction with the transcriptional regulator *SMAD9*. (JPG 322 kb)

Additional file 11 General linear model analysis testing for genotypic and allelic effects on cannon bone circumference. The effect on cannon bone circumference is shown for all three polymorphisms genotyped in 52 Shetland ponies whose cannon bone circumference measurements were available. (DOCX 13 kb)

Additional file 12 Primers and assays for validation of variants in ROH regions. SNVs in ROH regions specifically filtered for their functional effects were genotyped using Kompetitive Allele Specific PCR (KASP). Primer sequences, annealing temperature and number of PCR cycles are shown. (DOCX 23 kb)

Additional file 13 Primers for validation of two homozygous deletions found in Shetland ponies. Multiplex-PCR reverse primers located in the deletion and proximal of the deletion were used for targeting the two deletions. Product sizes and annealing temperatures are displayed. (DOCX 12 kb)

## Abbreviations

*ADAMTS17*: *Disintegrin-like and metalloprotease with thrombospondin type 1 motif 17*
*ANKRD1*: *Ankyrin repeat domain 1*
CNV: Copy number variant
*DIAPH3*: *Diaphanous related formin 3*
*FREM3*: *Related extracellular matrix 3*
*GAB1*: *Growth factor receptor bound protein 2-associated protein 1*
*GH1*: *Growth hormone 1*
GHR: Growth hormone receptor
GLM: General mixed model
*HMGA2*: *High mobility group AT-hook 2*
IGV: Integrative genomics viewer
*LASP1*: *LIM and SH3 protein 1*
*LCORL*: *Ligand dependent nuclear receptor corepressor like*
*MTMR10*: *Myotubularin related protein 10*
*MYO9A*: *Myosin IXA*
*OSTN*: *Osteocrin*
ROH: Runs of homozygosity
*SH3TC1*: *SH3 domain and tetratricopeptide repeats 1*
*SMARCA5*: *SWI/SNF related matrix associated actin dependent regulator of chromatin subfamily a member 5*
*SNTG1*: *Syntrophin gamma 1*
*TRPM1*: *Transient receptor potential cation channel subfamily M member 1*
*UTS2B*: *Urotensin 2B*
*VWA8*: *Von willebrand factor A domain containing 8*
*ZFAT*: *Zinc finger protein 406*
