# Supplementary material for: Genome data uncover four synergistic key regulators for extremely small body size in horses
Source: BMC Genomics. 2018 Jun 25;19:492. doi: 10.1186/s12864-018-4877-5 (PMC6019228; doi:10.1186/s12864-018-4877-5)
Supplement: Supplementary file 1 — Mapping statistics of whole-genome sequencing data. In total 32 samples of equids of different populations were analyzed in this study. Sequence read achieve ID, sequencing parameters and mean coverage are shown. (DOCX 15 kb) [file 12864_2018_4877_MOESM1_ESM.docx]

Additional file 1. Mapping statistics of whole-genome sequencing data. In total 32 samples of equids of different populations were analyzed in this study. Sequence read achieve ID, sequencing parameters and mean coverage are shown.

| Population (Sequence Read Achieve ID) | Platform | Number of lanes | Average read length | Bases mapped | Mean coverage (unmasked reference EquCab 2.0) |
| --- | --- | --- | --- | --- | --- |
| Duelmen Horse (SRX384479) | Illumina HiSeq 2000 | 1 | 97 | 37303793688 | 16.28 |
| Arabian  (SRX389472) | Illumina HiSeq 2000 | 1 | 97 | 35902587919 | 15.66 |
| Sorraia  (SRX389475) | Illumina HiSeq 2000 | 1 | 97 | 32924550979 | 14.37 |
| Hanoverian  (SRX389480) | Illumina HiSeq 2000 | 2 | 97 | 66783049097 | 29.14 |
| Hanoverian  (SRX389477) | Illumina HiSeq 2000 | 1 | 98 | 29105814366 | 12.70 |
| Hanoverian (SRX1131705) | Illumina HiSeq 2000 | 1 | 95 | 27809594069 | 12.13 |
| Hanoverian (SRX1131785) | Illumina HiSeq 2000 | 1 | 95 | 29381831812 | 12.82 |
| Saxon-Thuringian Heavy Warmblood (SRX1131818) | Illumina MiSeq | 4 | 246 | 38973433197 | 17.00 |
| Sorraia  (SRX1131820) | Illumina MiSeq | 4 | 244 | 34098144589 | 14.88 |
| Thoroughbred (SRX396629) | Illumina HiSeq 2000 | 1 | 94 | 39168236438 | 17.09 |
| Dokey  (SRS431817) | Illumina HiSeq 2000 | 1 | 65 | 46434952075 | 20.26 |
| Arabian  (SRS431663) | Illumina HiSeq 2000 | 1 | 95 | 94652196915 | 41.30 |
| Norwegian Fjord (SRS438157) | Illumina HiSeq 2000 | 1 | 95 | 82117206338 | 35.83 |
| Standardbred (SRS438330) | Illumina HiSeq 2000 | 1 | 86 | 88226580122 | 38.49 |
| Islandic Horse (SRS439179) | Illumina HiSeq 2000 | 1 | 94 | 133904496628 | 58.42 |
| Przewalski  (SRS441443) | Illumina HiSeq 2000 | 1 | 93 | 88071194708 | 38.43 |
| Marwari  (SRX535352) | Illumina HiSeq 2000 | 1 | 96 | 97348401623 | 42.47 |
| Connemara pony (SRX850674) | Illumina HiSeq 2000 | 1 | 98 | 15910875896 | 6.94 |
| Connemara pony (SRX850675) | Illumina HiSeq 2000 | 1 | 98 | 15302062299 | 6.68 |
| Miniature Shetland pony (SRX1976860) | Illumina NextSeq 500 | 1 | 145 | 41348536821 | 18.04 |
| Miniature Shetland pony (ERX947604) | Illumina HiSeq 2500 | 1 | 98 | 57571651352 | 25.12 |
| Shetland pony (ERX947605) | Illumina HiSeq 2500 | 1 | 99 | 63322857132 | 27.63 |
| Przewalski  (SRX305128) | Illumina HiSeq 2000 | 1 | 91 | 14558480418 | 6.35 |
| Przewalski  (SRX305127) | Illumina HiSeq 2000 | 1 | 92 | 23778310842 | 10.37 |
| Przewalski  (SRX302128) | Illumina HiSeq 2000 | 2 | 95 | 21725873643 | 9.48 |
| Przewalski  (SRX302111) | Illumina HiSeq 2000 | 2 | 95 | 19759467737 | 8.62 |
| Przewalski  (SRX302110) | Illumina HiSeq 2000 | 2 | 93 | 8137828887 | 3.55 |
| Scythian horse from Berel’  (SAMEA103910511) | Illumina HiSeq 2500 | 1 | 94 | 78384403219 | 34.20 |
| Scythian horse from Berel’  (SAMEA103910512) | Illumina HiSeq 2500 | 1 | 90 | 80712217505 | 35.22 |
| Scythian horse from Berel’  (SAMEA103910514) | Illumina HiSeq 2500 | 1 | 91 | 86296687398 | 37.65 |
| Scythian horse from Berel’  (SAMEA103910515) | Illumina HiSeq 2500 | 1 | 91 | 91509876772 | 39.93 |
| Scythian horse from Berel’  (SAMEA103910518) | Illumina HiSeq 2500 | 1 | 92 | 100145909621 | 43.69 |
