# Supplementary figures and images for: Genome data uncover four synergistic key regulators for extremely small body size in horses
Source: BMC Genomics. 2018 Jun 25;19:492. doi: 10.1186/s12864-018-4877-5 (PMC6019228; doi:10.1186/s12864-018-4877-5)

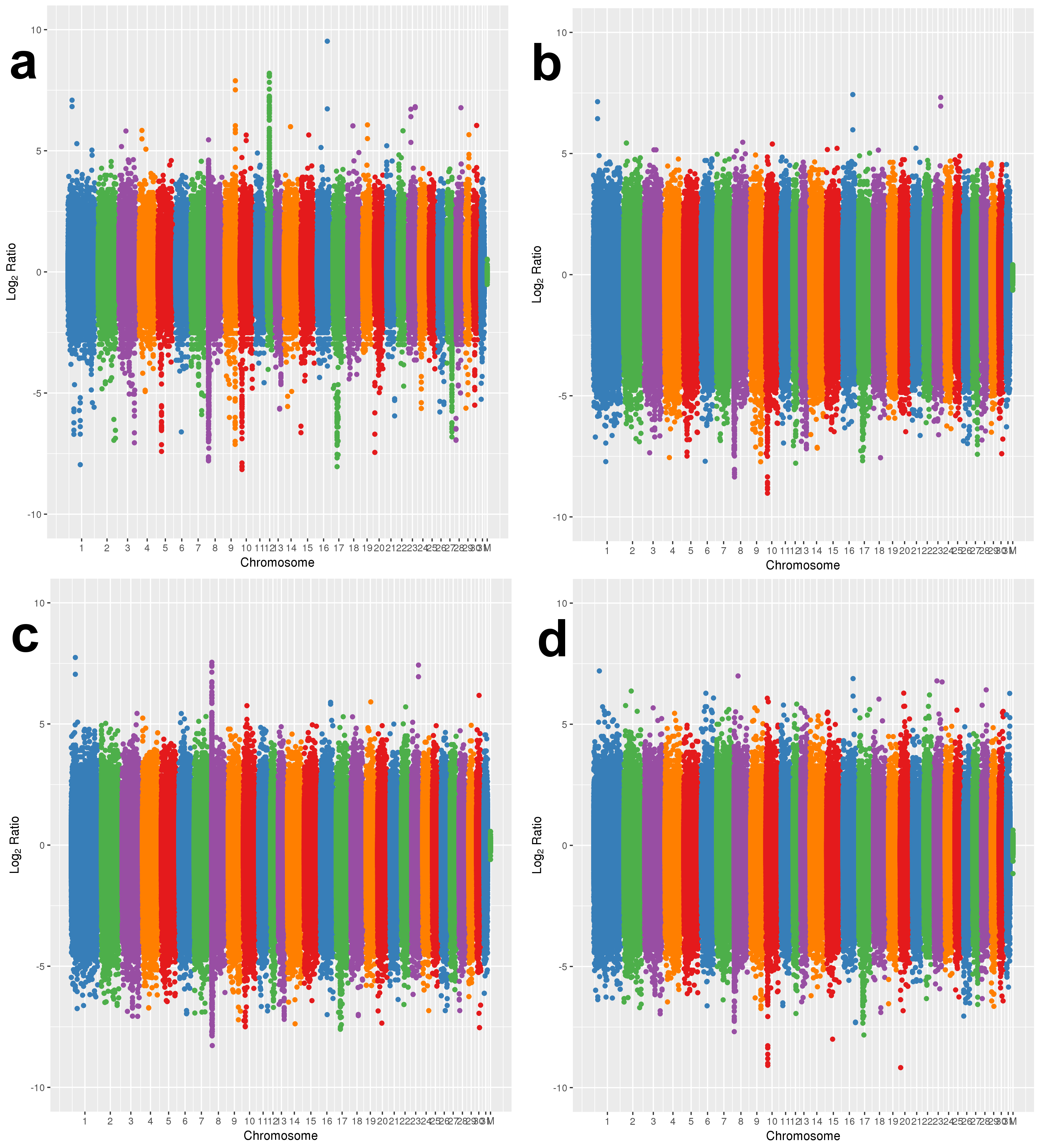

Supplement: Supplementary file 6 — Graphical representation of CNV detection results. Plots of log2 ratios of comparative analysis of three Shetland ponies with warmblood control group (a), Thoroughbred control group (b), non-breed control group (c) or “other breeds” control group (d) are displayed. (TIFF 3829 kb) [file 12864_2018_4877_MOESM6_ESM.tiff]

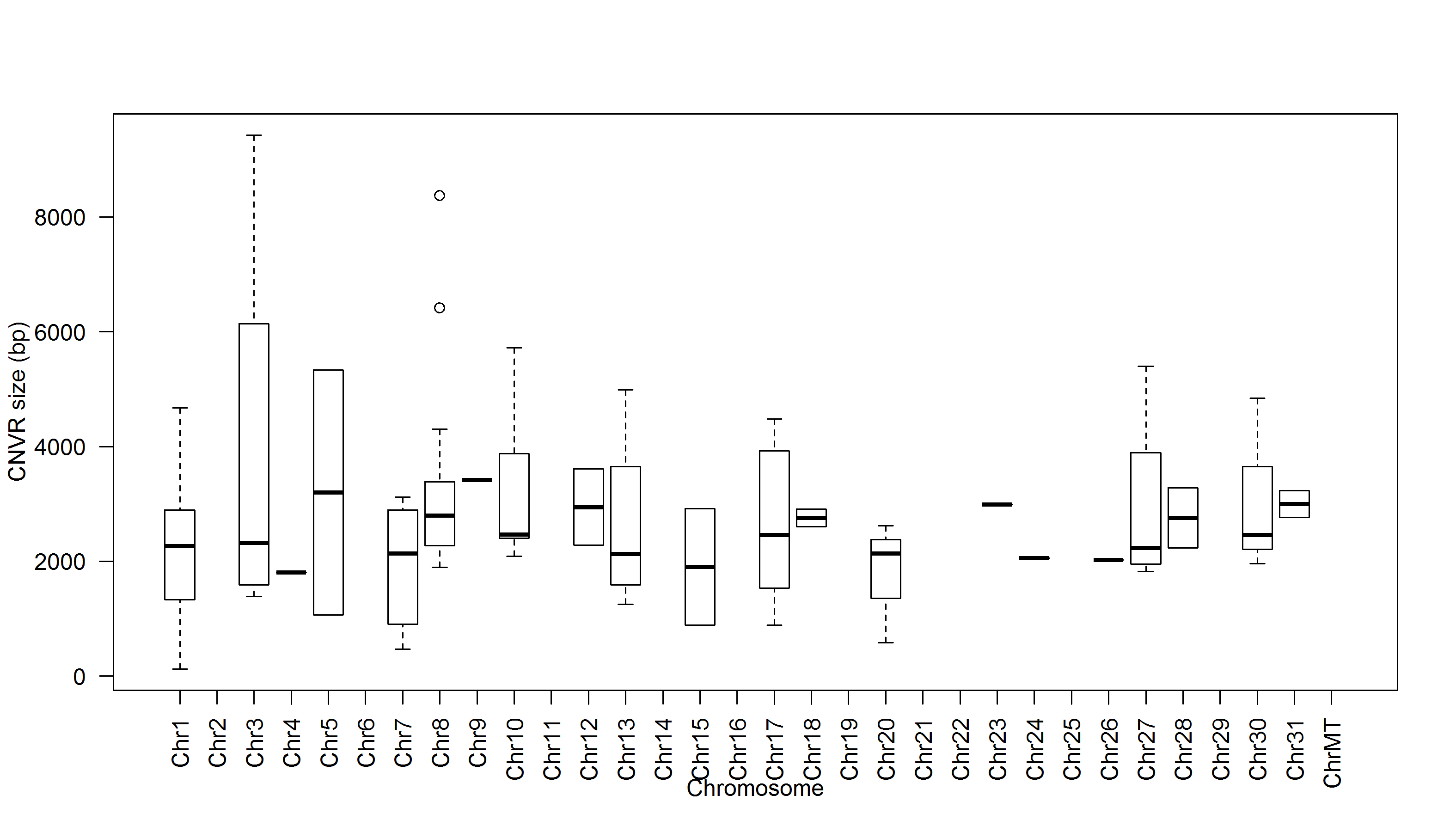

Supplement: Supplementary file 7 — Characterization of potential Shetland pony-specific CNV regions. The size of the 97 CNV regions identified in the intersection of all four CNV detection runs for Shetland pony-specific CNVs is shown in base pairs (bp) per chromosome. The number and size of all CNV regions varies among the chromosomes. On eleven chromosomes, no CNV region could be found in the overlapping results. (TIFF 16611 kb) [file 12864_2018_4877_MOESM7_ESM.tiff]

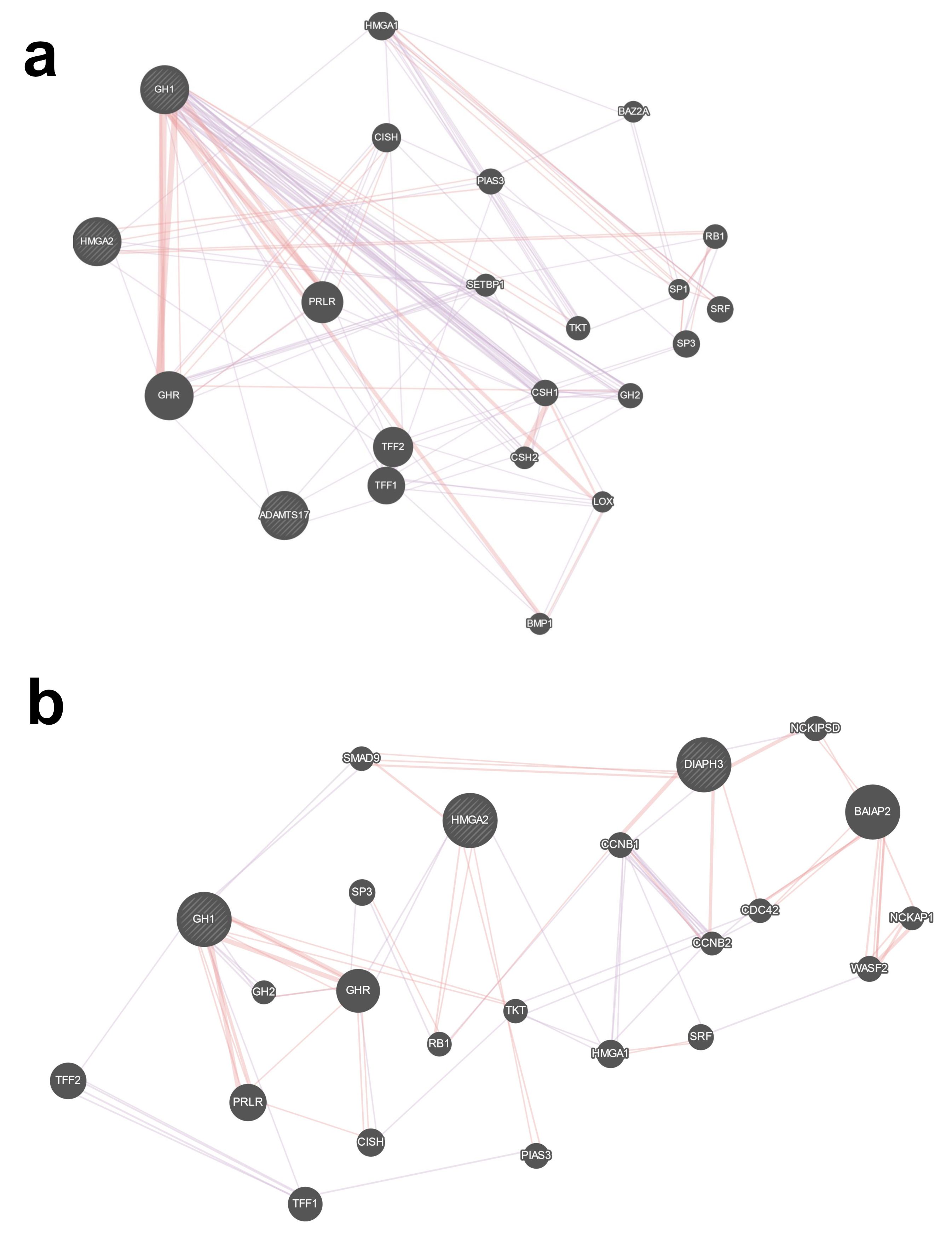

Supplement: Supplementary file 10 — Functional interaction network of candidate genes. Both, ADAMTS17 and HMGA2 show a co-expression (purple lines) with GHR, which is physically interacting (red lines) with GH1. Furthermore, ADAMTS17 is directly co-expressed with GH1. All three investigated genes GH1, HMGA2 and DIAPH3 show an interaction with the transcriptional regulator SMAD9. (JPG 322 kb) [file 12864_2018_4877_MOESM10_ESM.jpg]
