# Supplementary material for: Genome data uncover four synergistic key regulators for extremely small body size in horses
Source: BMC Genomics. 2018 Jun 25;19:492. doi: 10.1186/s12864-018-4877-5 (PMC6019228; doi:10.1186/s12864-018-4877-5)
Supplement: Supplementary file 8 — Intersection of potential Shetland pony-specific CNV regions (CNVRs). The overlap of four CNV detection results for Shetland pony-specific CNVRs shows 97 CNVR harboring 14 different genes. (DOCX 18 kb) [file 12864_2018_4877_MOESM8_ESM.docx]

Additional file 8. Intersection of potential Shetland pony-specific CNV regions (CNVRs). The overlap of four CNV detection results for Shetland pony-specific CNVRs shows 97 CNVR harboring 14 different genes.

| ECA | Start CNVR | End CNVR | Size | CNV type (cases) | CNVR location | Gene ID (Transcript ID) | Human orthologue |
| --- | --- | --- | --- | --- | --- | --- | --- |
| 1 | 24067462 | 24069705 | 2244 | loss | intergenic | - | *-* |
| 1 | 24073633 | 24076250 | 2618 | loss | intergenic | - | *-* |
| 1 | 24080411 | 24081799 | 1389 | loss | intergenic | - | *-* |
| 1 | 24092001 | 24093267 | 1267 | loss | intergenic | - | *-* |
| 1 | 24088593 | 24091049 | 2457 | loss | intergenic | - | *-* |
| 1 | 24094203 | 24097261 | 3059 | loss | intergenic | - | *-* |
| 1 | 24097261 | 24097381 | 121 | loss | intergenic | - | *-* |
| 1 | 24102261 | 24105048 | 2788 | loss | intergenic | - | *-* |
| 1 | 24106732 | 24110284 | 3553 | loss | intergenic | - | *-* |
| 1 | 24114421 | 24115520 | 1100 | loss | intergenic | - | *-* |
| 1 | 24111571 | 24114229 | 2659 | loss | intergenic | - | *-* |
| 1 | 24121879 | 24125057 | 3179 | loss | intergenic | - | *-* |
| 1 | 24129920 | 24132911 | 2992 | loss | intergenic | - | *-* |
| 1 | 24132912 | 24133561 | 650 | loss | intergenic | - | *-* |
| 1 | 24135981 | 24137979 | 1999 | loss | intergenic | - | *-* |
| 1 | 24141021 | 24143299 | 2279 | loss | intergenic | - | *-* |
| 1 | 24148246 | 24152920 | 4675 | loss | intergenic | - | *-* |
| 1 | 24156791 | 24158689 | 1899 | loss | intergenic | - | *-* |
| 1 | 158947541 | 158949629 | 2089 | loss | exonic and intronic | ENSECAG00000015131 (ENSECAT00000015793) | *TRAV20* |
| 1 | 166078273 | 166078807 | 535 | loss | intergenic | - | *-* |
| 3 | 71047285 | 71056711 | 9427 | loss | intergenic | - | *-* |
| 3 | 74816681 | 74819529 | 2849 | loss | intergenic | - | *-* |
| 3 | 115884943 | 115886331 | 1389 | loss | exonic and intronic | ENSECAG00000020879 (ENSECAT00000022463) | *SH3TC1* |
| 3 | 115897926 | 115899717 | 1792 | loss | intronic | ENSECAG00000020879 (ENSECAT00000022463) | *SH3TC1* |
| 4 | 96114635 | 96116439 | 1805 | gain | intergenic | - | *-* |
| 5 | 23565777 | 23566839 | 1063 | loss | intergenic | - | *-* |
| 5 | 23568447 | 23573781 | 5335 | loss | intergenic | - | *-* |
| 7 | 52727517 | 52730634 | 3118 | loss | intergenic | - | *-* |
| 7 | 65927997 | 65930701 | 2705 | loss | intergenic | - | *-* |
| 7 | 72204881 | 72207961 | 3081 | loss | exonic | ENSECAG00000008050 (ENSECAT00000008118) | *SSU72P8* |
| 7 | 72216970 | 72218999 | 2030 | loss | intergenic | - | *-* |
| 7 | 72220336 | 72222579 | 2244 | loss | intergenic | - | *-* |
| 7 | 72222767 | 72223369 | 603 | loss | intergenic | - | *-* |
| 7 | 73500361 | 73500829 | 469 | loss | intergenic | - | *-* |
| 7 | 73630591 | 73631801 | 1211 | loss | intergenic | - | *-* |
| 8 | 1619421 | 1622029 | 2609 | loss | intergenic | - | *-* |
| 8 | 1634194 | 1636469 | 2276 | loss | intergenic | - | *-* |
| 8 | 1638313 | 1641486 | 3174 | loss | intergenic | - | *-* |
| 8 | 1642585 | 1646261 | 3677 | loss | intergenic | - | *-* |
| 8 | 1658961 | 1661097 | 2137 | loss | intergenic | - | *-* |
| 8 | 1838959 | 1841859 | 2901 | loss | intergenic | - | *-* |
| 8 | 1866635 | 1869535 | 2901 | loss | exonic and intronic | ENSECAG00000014546 (ENSECAT00000015182) | *SLC7A4* |
| 8 | 2058310 | 2060529 | 2220 | loss | intergenic | - | *-* |
| 8 | 2076727 | 2079001 | 2275 | loss | intergenic | - | *-* |
| 8 | 2074766 | 2076727 | 1962 | loss | intergenic | - | *-* |
| 8 | 2093103 | 2095061 | 1959 | loss | intergenic | - | *-* |
| 8 | 2095951 | 2098327 | 2377 | loss | intergenic | - | *-* |
| 8 | 2181741 | 2185307 | 3567 | loss | exonic and intronic | ENSECAG00000016780  (ENSECAT00000017714) | *SLC7A4* |
| 8 | 2186909 | 2190291 | 3383 | loss | intergenic | - | *-* |
| 8 | 3878091 | 3880401 | 2311 | loss | intergenic | - | *-* |
| 8 | 3888589 | 3890481 | 1893 | loss | intergenic | - | *-* |
| 8 | 3896146 | 3899137 | 2992 | loss | intergenic | - | *-* |
| 8 | 3899325 | 3902117 | 2793 | loss | exonic | ENSECAG00000005220 (ENSECAT00000005096) | *IGLV1-51* |
| 8 | 4992901 | 4999309 | 6409 | loss | exonic | ENSECAG00000005614 (ENSECAT00000005534) | *IGLV8-61* |
| 8 | 4981865 | 4990231 | 8367 | loss | intergenic | - | *-* |
| 8 | 30251421 | 30255721 | 4301 | loss | intergenic | - | *-* |
| 9 | 69559951 | 69563369 | 3419 | loss | intergenic | - | *-* |
| 10 | 12431511 | 12433599 | 2089 | loss | intergenic | - | *-* |
| 10 | 12542861 | 12545263 | 2403 | loss | intergenic | - | *-* |
| 10 | 12547481 | 12553201 | 5721 | loss | intergenic | - | *-* |
| 10 | 12574321 | 12578193 | 3873 | loss | intergenic | - | *-* |
| 10 | 78010961 | 78013429 | 2469 | loss | intergenic | - | *-* |
| 12 | 14201551 | 14205159 | 3609 | gain | intergenic | - | *-* |
| 12 | 14213331 | 14215609 | 2279 | gain | intergenic | - | *-* |
| 13 | 40069581 | 40070827 | 1247 | loss | intergenic | - | *-* |
| 13 | 40066377 | 40068691 | 2315 | loss | intergenic | - | *-* |
| 13 | 40076345 | 40078281 | 1937 | loss | intergenic | - | *-* |
| 13 | 40085245 | 40090229 | 4985 | loss | intergenic | - | *-* |
| 15 | 28906601 | 28909515 | 2915 | loss | exonic and intronic | ENSECAG00000023365 (ENSECAT00000024987) | *C2ORF78* |
| 15 | 88097241 | 88098131 | 891 | loss | intergenic | - | *-* |
| 17 | 18817481 | 18818371 | 891 | loss | exonic and intronic | ENSECAG00000007666 (ENSECAT00000007686) | *NA* |
| 17 | 18921045 | 18923537 | 2493 | loss | intergenic | - | *-* |
| 17 | 18927206 | 18928361 | 1156 | loss | intergenic | - | *-* |
| 17 | 28375159 | 28377063 | 1905 | loss | intronic | ENSECAG00000006748 (ENSECAT00000008076) | *VWA8* |
| 17 | 28377117 | 28380855 | 3739 | loss | intronic | ENSECAG00000006748 (ENSECAT00000008076) | *VWA8* |
| 17 | 28384918 | 28389399 | 4482 | loss | intronic | ENSECAG00000006748 (ENSECAT00000008076) | *VWA8* |
| 17 | 35418241 | 35422357 | 4117 | loss | intronic | ENSECAG00000019161 (ENSECAT00000021056) | *DIAPH3* |
| 17 | 70174721 | 70177141 | 2421 | loss | intergenic | - | *-* |
| 18 | 16505229 | 16508141 | 2913 | loss | intergenic | - | *-* |
| 18 | 47405436 | 47408039 | 2604 | loss | intergenic | - | *-* |
| 20 | 39564417 | 39566549 | 2133 | loss | intergenic | - | *-* |
| 20 | 39569388 | 39569969 | 582 | loss | intergenic | - | *-* |
| 20 | 39573317 | 39575932 | 2616 | loss | intergenic | - | *-* |
| 23 | 48676101 | 48679092 | 2992 | loss | intergenic | - | *-* |
| 24 | 27138376 | 27140432 | 2057 | loss | intergenic | - | *-* |
| 26 | 40703261 | 40705281 | 2021 | loss | intergenic | - | *-* |
| 27 | 69777 | 72161 | 2385 | gain | intergenic | - | *-* |
| 27 | 37366921 | 37369009 | 2089 | gain | intronic | ENSECAG00000024680 (ENSECAT00000026863; ENSECAT00000026870; ENSECAT00000026873; ENSECAT00000026875; ENSECAT00000026877; ENSECAT00000017356) | *CSMD1; CSMD1; CSMD1; CSMD1; CSMD1; CSMD1; CSMD1* |
| 27 | 33647481 | 33649299 | 1819 | loss | intergenic | - | *-* |
| 27 | 33660513 | 33665909 | 5397 | loss | intergenic | - | *-* |
| 28 | 3017101 | 3020381 | 3281 | loss | intergenic | - | *-* |
| 28 | 44544941 | 44547171 | 2231 | loss | intergenic | - | *-* |
| 30 | 24611841 | 24616680 | 4840 | gain | exonic and intronic | ENSECAG00000006816 (ENSECAT00000006831; ENSECAT00000006979; ENSECAT00000006918) | *CFHR3/CFH; CFHR3/CFH; CFHR3/CFH* |
| 30 | 7998787 | 8000745 | 1959 | loss | intergenic | - | *-* |
| 30 | 9354271 | 9356732 | 2462 | loss | intergenic | - | *-* |
| 31 | 3697926 | 3701155 | 3230 | loss | intronic | ENSECAG00000004645 (ENSECAT00000029110) | *PRKN* |
| 31 | 3701333 | 3704096 | 2764 | loss | intronic | ENSECAG00000004645 (ENSECAT00000029110) | *PRKN* |
