# Supplementary material for: Genome data uncover four synergistic key regulators for extremely small body size in horses
Source: BMC Genomics. 2018 Jun 25;19:492. doi: 10.1186/s12864-018-4877-5 (PMC6019228; doi:10.1186/s12864-018-4877-5)
Supplement: Supplementary file 9 — Genetic effects for height at the withers. The individual additive effects of all four candidate SNPs and the CNV located in DIAPH3 as well as simultaneous testing results for additive and dominant effects are shown. (DOCX 13 kb) [file 12864_2018_4877_MOESM9_ESM.docx]

Additional file 9. Additive and dominant genetic effects for height at the withers. Single SNP testing for additive effects of all four candidate SNPs as well as simultaneous testing results for additive and dominant effects are shown.

| ECA | Gene | Polymorphism | Estimate | Standard error | t value | P-value |
| --- | --- | --- | --- | --- | --- | --- |
| Single SNP testing - additive effects | | |  |  |  |  |
| 1 | *ADAMTS17* | NC_009144.2:g.105258161C>A | -7.933 | 0.779 | -10.19 | <0.0001 |
| 6 | *HMGA2* | c.83G>A | -4.262 | 0.780 | -5.47 | <0.0001 |
| 19 | *OSTN* | NC_009144.2:g.28594461G>A | -7.405 | 0.757 | -9.79 | <0.0001 |
| 11 | *GH1* | NC_009144.2:g.15520392C>T | -6.872 | 1.013 | -6.79 | <0.0001 |
| Simultaneously in one model - additive effects | | |  |  |  |  |
| 1 | *ADAMTS17* | NC_009144.2:g.105258161C>A | -6.166 | 0.703 | -8.76 | <0.0001 |
| 6 | *HMGA2* | c.83G>A | -4.646 | 0.501 | -9.27 | <0.0001 |
| 19 | *OSTN* | NC_009144.2:g.28594461G>A | -3.620 | 0.685 | -5.28 | <0.0001 |
| 11 | *GH1* | NC_009144.2:g.15520392C>T | -4.052 | 0.643 | -6.30 | <0.0001 |
| Simultaneously in one model - dominant effects | | |  |  |  |  |
| 1 | *ADAMTS17* | NC_009144.2:g.105258161C>A | 1.239 | 1.001 | 1.24 | 0.2171 |
| 6 | *HMGA2* | c.83G>A | -1.853 | 1.812 | -1.02 | 0.3075 |
| 19 | *OSTN* | NC_009144.2:g.28594461G>A | -3.211 | 0.963 | -3.33 | 0.0010 |
| 11 | *GH1* | NC_009144.2:g.15520392C>T | -0.891 | 1.018 | -0.88 | 0.3822 |
