# Supplementary material for: Genome data uncover four synergistic key regulators for extremely small body size in horses
Source: BMC Genomics. 2018 Jun 25;19:492. doi: 10.1186/s12864-018-4877-5 (PMC6019228; doi:10.1186/s12864-018-4877-5)
Supplement: Supplementary file 11 — General linear model analysis testing for genotypic and allelic effects on cannon bone circumference. The effect on cannon bone circumference is shown for all three polymorphisms genotyped in 52 Shetland ponies whose cannon bone circumference measurements were available. (DOCX 13 kb) [file 12864_2018_4877_MOESM11_ESM.docx]

Additional file 11. General linear model analysis testing for genotypic and allelic effects on cannon bone circumference. The effect on cannon bone circumference is shown for all three polymorphisms genotyped in 52 Shetland ponies whose cannon bone circumference measurements were available.

| ECA | Gene | Polymorphism | R-Square | F-value | P-value | Genotype | LSMean (cm) | Standard error | |
| --- | --- | --- | --- | --- | --- | --- | --- | --- | --- |
| Genotype effect | | |  |  |  |  |  |  | |
| 1 | *ADAMTS17* | NC_009144.2:g.105258161C>A | 0.01 | 0.44 | 0.51155 | A/A | 13.46 | 0.23 | |
|  |  |  |  |  |  | C/A | 14.00 | 0.79 | |
|  |  |  |  |  |  | C/C | - | - | |
| 6 | *HMGA2* | c.83G>A | 0.52 | 26.15 | 1.8689E-8 | G/G | 15.42 | 0.32 | |
|  |  |  |  |  |  | G/A | 11.80 | 0.50 | |
|  |  |  |  |  |  | A/A | 13.09 | 0.19 | |
| 19 | *OSTN* | NC_009144.2:g.28594461G>A | 0.12 | 3.41 | 0.041183 | A/A | 13.34 | 0.22 | |
|  |  |  |  |  |  | G/A | 14.63 | 0.75 | |
|  |  |  |  |  |  | G/G | 16.50 | 1.50 | |
| 11 | *GH1* | NC_009144.2:g.15520392C>T | 0.19 | 5.85 | 0.005263 | C/C | 13.97 | 0.25 | |
|  |  |  |  |  |  | C/T | 12.96 | 0.38 | |
|  |  |  |  |  |  | T/T | 11.90 | 0.64 | |
| Allele effect | | | | | | | | |  |
| 1 | *ADAMTS17* | NC_009144.2:g.105258161C>A | 0.004 | 0.43 | 0.51538 | A | 13.48 | 0.16 |  |
|  |  |  |  |  |  | C | 14.00 | 0.78 |  |
| 6 | *HMGA2* | c.83G>A | 0.15 | 17.58 | 0.000059 | G | 14.35 | 0.25 |  |
|  |  |  |  |  |  | A | 13.09 | 0.17 |  |
| 19 | *OSTN* | NC_009144.2:g.28594461G>A | 0.08 | 8.63 | 0.004091 | A | 13.39 | 0.15 |  |
|  |  |  |  |  |  | G | 15.25 | 0.61 |  |
| 11 | *GH1* | NC_009144.2:g.15520392C>T | 0.12 | 13.86 | 0.000322 | C | 13.79 | 0.16 |  |
|  |  |  |  |  |  | T | 12.52 | 0.30 |  |
