# Supplementary material for: Genome data uncover four synergistic key regulators for extremely small body size in horses
Source: BMC Genomics. 2018 Jun 25;19:492. doi: 10.1186/s12864-018-4877-5 (PMC6019228; doi:10.1186/s12864-018-4877-5)
Supplement: Supplementary file 12 — Primers and assays for validation of variants in ROH regions. SNVs in ROH regions specifically filtered for their functional effects were genotyped using Kompetitive Allele Specific PCR (KASP). Primer sequences, annealing temperature and number of PCR cycles are shown. (DOCX 23 kb) [file 12864_2018_4877_MOESM12_ESM.docx]

Additional file 12. Primers and assays for validation of variants in ROH regions. SNVs in ROH regions specifically filtered for their functional effects were genotyped using Kompetitive Allele Specific PCR (KASP). Primer sequences, annealing temperature and number of PCR cycles are shown.

| ECA | Location | Base change | Primer marked with FAM or VIC (5’-3’) | Common primer (5’-3’) | Annealing temperature (°C) | Number of cycles |
| --- | --- | --- | --- | --- | --- | --- |
| 1 | 105258161 | C/A | GCCAGGACTGTTTGTCCATCTG-FAM  GGCCAGGACTGTTTGTCCATCTT-VIC | ACCCACCTGTGACCACTCGGAT | 61 | 29 |
| 9 | 36855980 | T/A | ATTTTTTCTGAGAGTCAGGAATTAAGTTCT-FAM  TTTTCTGAGAGTCAGGAATTAAGTTCA-VIC | CKGGAACTTGTGTTGTTGATGTAGGAA | 61 | 29 |
| 11 | 15475283 | T/C | CACTCAGGCAGGGATATGAGTGA-FAM  ACTCAGGCAGGGATATGAGTGG-VIC | CAGCGCCATCCAAGGTGCTCAT | 61 | 26 |
| 11 | 15520392 | C/T | GGCGTTCTTTCTCCCAGGAGC-FAM  GGCGTTCTTTCTCCCAGGAGT-VIC | GAGTATCTCTGTCCCTCGGGGAT | 61 | 29 |
| 11 | 15849785 | G/C | AGAGCTGCCGCCCCGGG-FAM  AGAGCTGCCGCCCCGGC-VIC | MACTCGACCCCGCGCTGCA | 61 | 29 |
| 19 | 28594461 | G/A | GTTTCTTCTCCAAGAGTTCCTTACC-FAM  CGTTTCTTCTCCAAGAGTTCCTTACT-VIC | GCTGGCTCTCTAGAGCATAAAGGTA | 61 | 29 |
| 28 | 7301507 | C/T | GTACCTTTTTAAAGTCAGTTGAGTCATC-FAM  AAGTACCTTTTTAAAGTCAGTTGAGTCATT-VIC | AGRAAACCTCACGGTAGCAGGCTA | 61 | 29 |
| 28 | 7329353 | C/G | AGAACTAATCAAATCATTATGTCTTCTACC-FAM  AGAACTAATCAAATCATTATGTCTTCTACG-VIC | GATATGACTCTTTCCTTTAGTTGCTCCTT | 61 | 26 |
