# Supplementary material for: Genome data uncover four synergistic key regulators for extremely small body size in horses
Source: BMC Genomics. 2018 Jun 25;19:492. doi: 10.1186/s12864-018-4877-5 (PMC6019228; doi:10.1186/s12864-018-4877-5)
Supplement: Supplementary file 13 — Primers for validation of two homozygous deletions found in Shetland ponies. Multiplex-PCR reverse primers located in the deletion and proximal of the deletion were used for targeting the two deletions. Product sizes and annealing temperatures are displayed. (DOCX 12 kb) [file 12864_2018_4877_MOESM13_ESM.docx]

Additional file 13. Primers for validation of two homozygous deletions found in Shetland ponies. Multiplex-PCR reverse primers located in the deletion and proximal of the deletion were used for targeting the two deletions. Product sizes and annealing temperatures are displayed.

| Gene | Forward primer (5’-3’) | Reverse primer (5’-3’) | Product size (bp) | Annealing temperature (°C) |
| --- | --- | --- | --- | --- |
| *VWA8* | ATCTCTTTGCTCAAGGCTCTG | CCCCTATCAGGTATTCAGTGG | 288 | 58.5 |
| *VWA8* | ATCTCTTTGCTCAAGGCTCTG | CACTATGCGAAGACACCAATC | 1022 | 58.5 |
| *DIAPH3* | AATTCAAGATTATTGGCTTTGC | GTCTACCTTTTCACGTTTCCAC | 257 | 58.5 |
| *DIAPH3* | AATTCAAGATTATTGGCTTTGC | TTTTCTTCTTCAGGTCCTCTTG | 825 | 58.5 |
